# Land transpiration-evaporation partitioning errors responsible for modeled summertime warm bias in the central United States

**DOI:** 10.1038/s41467-021-27938-6

**Published:** 2022-01-17

**Authors:** Jianzhi Dong, Fangni Lei, Wade T. Crow

**Affiliations:** 1 USDA ARS Hydrology and Remote Sensing Laboratory, Beltsville, MD USA; 2 Institute of Surface-Earth System Science, Tianjin University, Tianjin, China; 3 Geosystems Research Institute, Mississippi State University, Starkville, MS USA; 4 Present Address: Department of Civil and Environmental Engineering, Massachusetts Institute of Technology, Cambridge, MA USA

**Keywords:** Hydrology, Hydrology

## Abstract

Earth system models (ESMs) from the Coupled Model Intercomparison Project Phase 6 (CMIP6) experiment exhibit a well-known summertime warm bias in mid-latitude land regions – most notably in the central contiguous United States (CUS). The dominant source of this bias is still under debate. Using validated datasets and both coupled and off-line modeling, we find that the CUS summertime warm bias is driven by the incorrect partitioning of evapotranspiration (ET) into its canopy transpiration and soil evaporation components. Specifically, CMIP6 ESMs do not effectively use available rootzone soil moisture for summertime transpiration and instead rely excessively on shallow soil and canopy-intercepted water storage to supply ET. As such, expected summertime precipitation deficits in CUS induce a negative ET bias into CMIP6 ESMs and a corresponding positive temperature bias via local land-atmosphere coupling. This tendency potentially biases CMIP6 projections of regional water stress and summertime air temperature variability under elevated CO_2_ conditions.

## Abstract (web summary)

Summertime warm bias in the central United States persists in Earth System Models. This bias is dominated by land physics related to transpiration and evaporation partitioning. Improved land physics can constrain projected climate uncertainty.

## Introduction

Global warming leads to an increased risk of local hydroclimate extremes, e.g., heatwaves^1–3^, droughts^4–7^, and floods^8–10^. Earth System Models (ESMs) are commonly applied to project such risks^11–14^. However, retrospective ESMs are often biased with regard to regional screen-level air temperature (T_2m_)—particularly during mid-latitude summer (June–July–August or JJA)^15^. Understanding the process-level source of T_2m_ bias has been a priority for the Coupled Model Intercomparison Project Phase 6 (CMIP6) experiment^16^. Due to the importance of mid-latitude JJA conditions for food production^17^, resolving this bias would significantly benefit the credibility of ESM projections as the basis for climate adaptation strategies. Positive JJA T_2m_ bias is most notable in the central contiguous United States (CUS) and remains a persistent feature there in both coupled ESMs and numerical weather prediction models—even after multiple generations of model development^15,18^.

The CUS JJA warm bias is potentially attributable to either ESM atmosphere or land surface modeling errors, and the dominant contributor to such bias is still under debate. For instance, most ESMs cannot capture the summer nocturnal precipitation (P) peak in CUS, which can result in negatively biased JJA P^19^. Such diurnal P bias is potentially attributable to the incorrect representation of convective-system propagation^20–22^ and the low-level jet in the CUS^23–25^. Errors in these processes also lead to underestimated shallow cumulus cloud cover and overestimated net shortwave solar radiation (Rs)^18^. The net impact of negatively biased P and positively biased Rs eventually yields excessive surface sensible heating^26,27^ and an evapotranspiration (ET) deficit^18,26,28,29^. Based on this line of reasoning, attention has generally been focused on isolating atmospheric sources for the CUS JJA warm bias.

However, even in the absence of bias in atmospheric variables, internal shortcomings in ESM land surface physics can significantly impact the surface partitioning of sensible and latent heat flux^30^ and, by extension, JJA T_2m_ levels through local land-atmosphere coupling. In off-line land surface models (LSMs), employing different soil moisture (SM) stress functions can lead to relative ET differences of up to 50%^31,32^. Likewise, the transpiration component of ET is particularly sensitive to the representation of lateral soil water flow between model grid cells^33^ and/or groundwater dynamics^34^. Due to their general inability to represent these processes, LSMs tend to overestimate the incidence of water-limited ET regimes^35^—and therefore overestimate T_2m_ when applied in a coupled ESM^36,37^.

As mentioned above, a low bias in ET can be attributed to either atmospheric (e.g., negatively biased P^18^) or land (e.g., an over-coupled SM–ET relationship^32^) modeling errors. Previous studies have not disentangled competing atmospheric and land-modeling errors and, as such, have not isolated the key factor(s) controlling the CUS warm bias in ESMs. Although experiments involving off-line LSM simulations are helpful in identifying land-based biases, their findings cannot be directly transferred to coupled ESMs—since the relative importance of atmospheric and land-modeling errors is unknown, and land biases tend to be attenuated after considering land-atmosphere feedbacks^38^. Therefore, the conclusive attribution of the CUS JJA warm bias is challenged by the complex relationship between the land and the atmosphere.

Fortunately, recent advances in remote sensing (RS) have significantly improved the accuracy of retrieved land variables^39–42^. New statistical approaches are also available that provide unbiased estimates of land-atmosphere coupling strength using (relatively) noisy RS retrievals^43^. In addition, newly available off-line LSM simulations forced with observed meteorological observations, but utilizing the same land physics as CMIP6 simulations, can help separate land and atmospheric contributions to CMIP6 T_2m_ biases^44^. Utilizing these advances, we investigate JJA T_2m_ biases within the latest CMIP6 ESMs for the CUS region and identify key variables/processes that control CMIP6 air temperature bias and model-to-model variance.

Our results demonstrate that the CUS JJA warm bias in CMIP6 ESMs is attributable to the incorrect partitioning of ET into its canopy transpiration and soil evaporation components. This partitioning bias between different ET components is related to the inadequate utilization of available rootzone SM (RZSM) for ET in ESMs. As a result, CMIP6 ESMs are disproportionally impacted by seasonal variations in P and generate negatively biased summertime ET estimates that propagate into a warm JJA bias via local land-atmosphere coupling. Biased land partitioning of transpiration versus evaporation also tends to yield overestimated JJA temperature variability under future elevated CO_2_ conditions.

## Results

This section separates potential sources of the warm CUS JJA bias and quantifies their relative importance. Specifically, we consider: (1) a Rs-dominant hypothesis where a positive bias in Rs introduces a corresponding positive T_2m_ bias via excessive surface sensible heating associated with depleted RZSM; (2) a P-dominant hypothesis where a negative bias in P directly leads to underestimated RZSM and excessive surface sensible heat flux; and (3) a land-dominant hypothesis where incorrect land physics yield a negative bias in JJA ET that outweighs the impact of P and Rs biases. Since modeled T_2m_ bias is most evident in 2-m summertime daily maximum temperature (Tmax)^27^, we focus on the analysis of Tmax bias. Given that ET estimates from FluxCOM^45^ have the least absolute bias among available ET reference products relative to ground-based flux-tower observations (Supplementary Fig. 1), they are applied here as an ET reference.

**Fig. 1:**
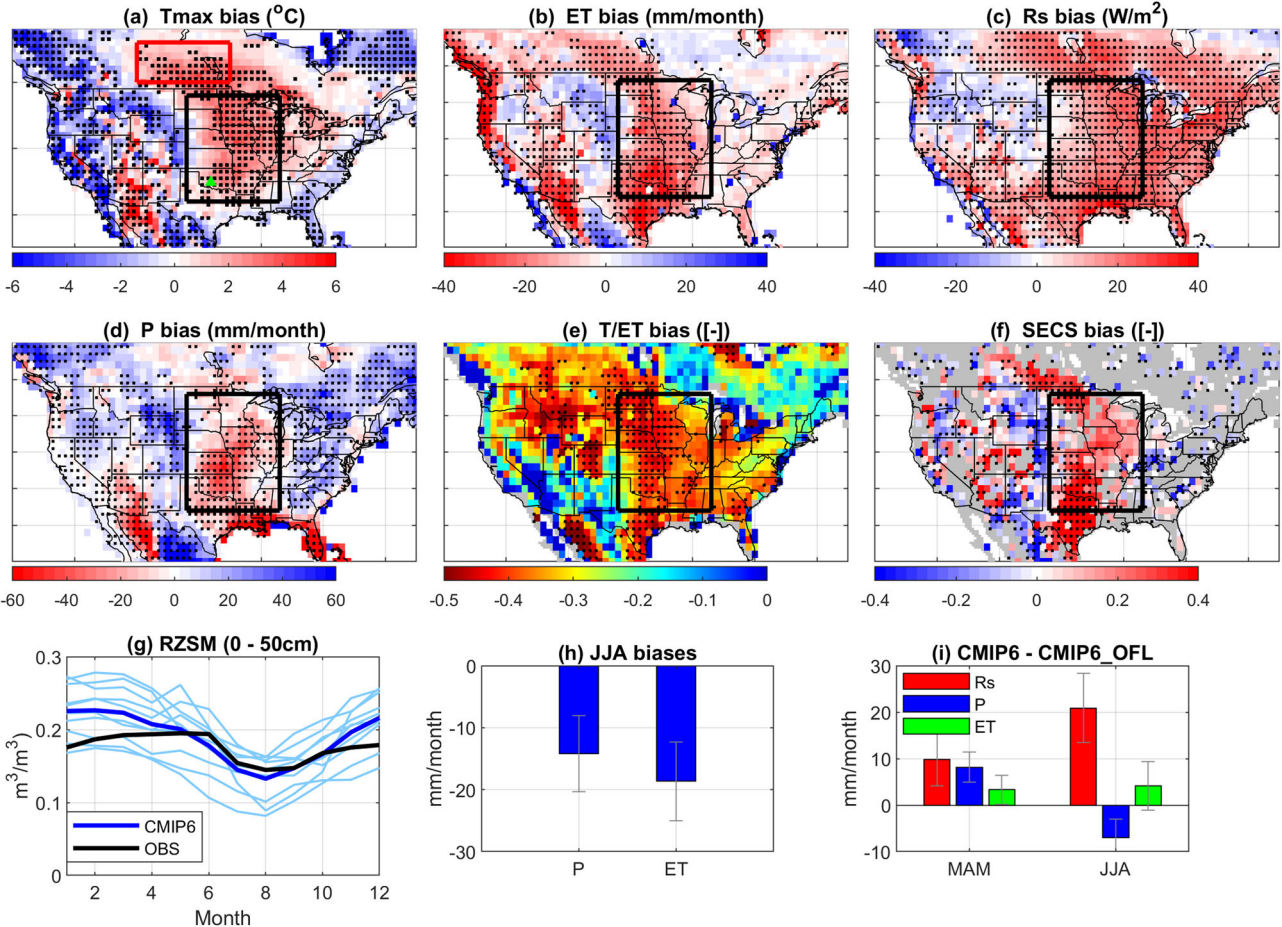
Spatial distribution and temporal evolution of CMIP6 biases. 2000–2014 CMIP6 biases within JJA (June-July-August): Tmax (2-m summertime daily maximum temperature, **a**), evapotranspiration (ET, **b**), net shortwave radiation (Rs, **c**), precipitation (P, **d**), Global Land Evaporation Amsterdam Model (GLEAM) evaluated transpiration (T) to ET ratio (T/ET, **e**) and soil moisture—ET coupling strength (SECS, **f**) estimates. Dotted grids indicate that two-thirds of individual Earth System Model (ESM) biases are consistent in sign (in (**a**)–(**d**) and (**f**)) or two-thirds of ESMs are 0.3 lower than GLEAM-based T/ET (in (**e**)). **g** Monthly mean CMIP6 (Coupled Model Intercomparison Project Phase 6) RZSM (rootzone soil moisture) estimates compared to a dense soil moisture network (OBS) within the state of Oklahoma (see the green triangle in part **a**). The thin and thick blue lines represent individual ESMs and their multi-model medians, respectively. **h** June–July–August (JJA) CMIP6 P and ET biases averaged across 15 CMIP6 ESMs. Error bars denote their inter-model standard deviations. **i** The mean difference of CMIP6 and CMIP6_OFL (CMIP6 off-line simulations) based Rs, P, and ET estimates for three individual ESMs during both March-April-May (MAM) and JJA. Error bars represent the range of CMIP6 and CMIP6_OFL differences. All ET bias results use FluxCOM as a baseline reference; however, no qualitative change is found when a different ET reference is used instead (see Supplementary Fig. 4).

Consistent with previous CMIP3 and CMIP5 analyses^15,18^, retrospective coupled CMIP6 results demonstrate a warm Tmax bias in the CUS region (Fig. 1a) that extends into southern Canada. Spatial comparisons show that 72.7% of CUS grid cells with significant Tmax warm biases also contain significant negative ET biases.

The net impact of large-scale temperature advection is a relatively small contributor to total diurnal heating of the lower atmosphere in the CUS region (see Supplementary Fig. 2). In addition, the hypothesis that the Tmax bias is dominated by ESM errors in temperature advection is inconsistent with simple surface water balance considerations (see Section 3 of the supporting information). As a result, this study focuses on the role of local processes.

### Rs-dominant hypothesis

As expected, Fig. 1c shows that CMIP6 Rs estimates have a clear positive bias throughout the CUS region. Overestimated Rs can potentially yield negatively biased ET via the depletion of RZSM. However, if this mechanism is truly the dominant source of JJA ET and Tmax biases, CMIP6 ESMs should predict excessively low RZSM during JJA—irrespective of whether the Rs impact on RZSM is inter- or intra-seasonal in nature. To evaluate this possibility, we compare seasonal trends in 2000–2014 CMIP6 JJA RZSM estimates to comparable results from a dense SM network in Oklahoma (see the green triangle in Fig. 1a)—an area where at least two-thirds of CMIP6 ESMs demonstrate significant biases in Tmax and ET (Fig. 1a, b). As shown in Fig. 1g, CMIP6 ESMs encompass the observed RZSM seasonal cycle and there is no evidence of biased RZSM depletion during JJA. Likewise, paired *T*-test results based on an additional 27 sparse SM in-situ sites in the CUS region also demonstrate that the difference of CMIP6- and observation-based May-JJA RZSM drying rate is statistically insignificant (at *p* = 0.95 confidence level, Supplementary Fig. 3). Figure 1h further evaluates potential RZSM deficits from a water balance perspective. It shows that the total (negative) ET bias is substantially stronger than the accompanying negative P bias—which illustrates that CUS JJA water availability (i.e., P-ET) is generally overestimated in CMIP6 ESMs. Therefore, from both a RZSM and water balance perspective, there is no support for the hypothesis that a positive bias in Rs induces a dry JJA RZSM bias. The lack of an apparent bias in CMIP6 RZSM estimates suggests that observed Tmax and ET biases cannot be explained via a JJA RZSM deficit in the CMIP6 ESMs. These results also argue against any hypothesis invoking excessive springtime model RZSM depletion due to intra- or inter-seasonal land and/or atmosphere modeling errors, e.g., biases in snow albedo modeling^46^ or excessive spring drying due to phenological changes^47^.

Coupled and off-line (with the same land physics but observation-corrected meteorological forcing data, denoted as CMIP6_OFL) CMIP6 simulations are compared to further examine the Rs-dominate hypothesis. Rs and ET relative differences between the CMIP6 and CMIP6_OFL cases (Fig. 1i) suggest that the overestimation of Rs in the coupled CMIP6 simulations generally increases JJA ET via increased atmospheric ET demand—a tendency that overwhelms any potential decrease in ET related to RZSM depletion. Therefore, there is no indication that a positive Rs bias (present in the CMIP6 simulations—but corrected for in CMIP6_OFL) is contributing to the negative JJA ET bias seen in Fig. 1b.

### P-dominant hypothesis

Spatial analysis is useful for examining the (second) potential hypothesis that underestimated P is the dominant source of ET and Tmax biases. Figure 1d illustrates that significant JJA P bias is concentrated in the southern CUS. However, in the northern CUS (i.e., roughly the northern third of the black box in Fig. 1a) and areas of southern Canada (i.e., the red box in Fig. 1a), regional Tmax and ET biases persist (at levels up to 3 °C and 20 W/m^2^, respectively) in the absence of any significant P bias (compare Fig. 1a, d).

Likewise, if a negative P bias is the primary contributor to the ET deficit and warm Tmax bias, summertime drying trends in CUS RZSM should be overestimated by the CMIP6 ESMs. Instead, as discussed above, no significant CMIP6 CUS JJA RZSM drying biases can be identified based on independent comparisons to either in-situ RZSM measurements or P-ET observations (see Fig. 1g, h). This suggests that observed CUS ET and Tmax biases are unlikely to originate from underestimated P. This finding holds even when intra-seasonal P bias (i.e., the lagged impact of a spring P deficit on summer ET via long-term SM memory) is considered (Supplementary Fig. 4).

**Fig. 2:**
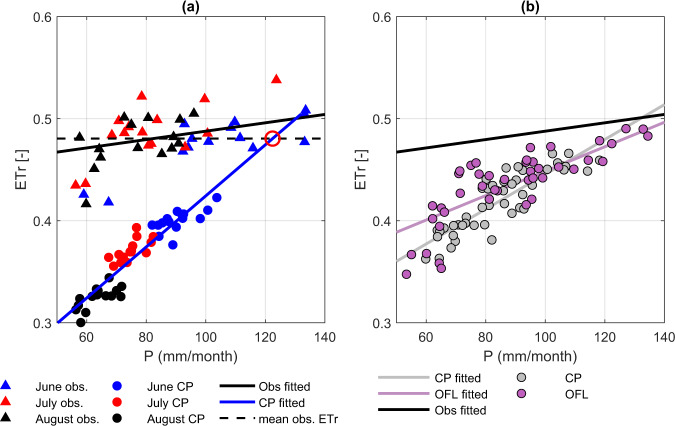
Sensitivity of CMIP6-modeled ET to reduced precipitation. **a** Observed and CMIP6 (Coupled Model Intercomparison Project Phase 6) modeled CUS (central contiguous United States) monthly mean ETr (calculated as the ratio of evapotranspiration (ET) and net shortwave ration (Rs), or ET/Rs) as a function of monthly mean precipitation (P) during 2000–2014 June–July–August (JJA) periods. The large open red circle in (**a**) captures the intersection of the extrapolated CMIP6 regression line with the monthly mean-observed ETr line. Part (**b**) is same as (a) but for 3-model-averaged (EC-Earth3-Veg, IPSL-CM6A-LR and MIROC6) CMIP6 (denoted as CP) and CMIP6_OFL (CMIP6 off-line simulation, denoted as OFL) results. ET values are based on FluxCOM estimates; however, no qualitative change is found when a different ET reference is used instead (see Supplementary Fig. 5).

Figure 2a provides additional insight into the impact of a P deficit on CMIP6 ET bias. To isolate inter-month and inter-model Rs variability, monthly ET are normalized by monthly Rs averages (and denoted as ETr) to reflect the fraction of Rs converted into ET. The sensitivity (i.e., slope) of ETr to P seen in the CMIP6 ESMs is substantially higher than the observed relationship—meaning that land models imbedded in CMIP6 simulations are biased in their surface energy flux partitioning for a given monthly P. As a result of this enhanced sensitivity, the observed bias in CMIP6 ETr increases as P seasonally decreases from June to August in the CUS. The magnitude of this sensitivity bias indicates that, although increased P can alleviate the ETr and Tmax biases, it cannot entirely remove the CUS warm/dry bias in a physically plausible manner. For instance, the regression line of CMIP6-based P and ETr suggests that to achieve observed ETr levels without modifying CMIP6 land surface physics requires an average of ~60 mm/month additional P, which is 44% higher than observed P. Notably, this finding is also qualitatively reflected in CMIP6_OFL simulations based on relatively unbiased meteorological forcing (Fig. 2b).

The above analyses demonstrate that CMIP6 ET bias, and the associated excessive sensitivity of ETr to P, is primarily attributable to land-modeling errors that cannot be addressed solely through the correction of P bias. Although the ETr-P relationship varies across different ESMs, our findings are applicable to individual ESMs, except for MRI-ESM2-0, which generally exhibits the smallest ET and Tmax biases across all ESMs (Supplementary Fig. 6).

### Land-dominant hypothesis

Above, we demonstrate that CUS JJA Tmax and ET biases are poorly explained by corresponding biases in P and Rs—leading to the hypothesis that errors in land physics dominate the CUS warm/dry JJA bias in CMIP6 simulations. According to the gauge-based CPC precipitation and FluxCOM datasets, CUS JJA ET is 29% higher than P—suggesting that a substantial amount of JJA ET is supplied from RZSM accumulated during the spring. This leads us to the hypothesis that, due to their biased land physics, CMIP6 land models do not effectively use available RZSM storage for JJA transpiration (T)—which subsequently leads to negatively biased ET and eventually translates into positively biased Tmax via local land-atmosphere coupling. As such, insufficient use of RZSM ensures that CMIP6 ESMs overestimate T water stress and rely excessively on soil evaporation to supply ET (i.e., a negative bias in T/ET), which eventually yields a warm Tmax bias. The negative bias of CMIP6-estimated T/ET over the CUS regions is identifiable from three independent perspectives.

First, direct comparisons against GLEAM-based T and ET data confirm that CMIP6 ESMs generally underestimate T/ET bias in the CUS region (Fig. 1e)—reflecting the general inability of ESMs to capture the complex interactions of vegetation, RZSM^48^ and climate over dry-wet transitional zones (Supplementary Fig. 7). It should be noted that the negative bias in T/ET for most ESMs exceeds 0.3 in the CUS region. Therefore, although GLEAM-based T/ET estimates undoubtably contain their own biases, our finding that CMIP6 generally underestimates CUS T/ET is qualitatively robust unless the GLEAM T/ET bias exceeds 0.3.

Second, as noted above, CMIP6-modeled ETr is overly sensitive to seasonal P variations (Fig. 2a), and this excess sensitivity cannot be attributed to meteorological forcing biases (Fig. 2b). From water balance considerations, the positive bias in ETr to P sensitivity suggests that CMIP6-estimated ET relies excessively on P-related fast water storages (e.g., surface SM and canopy-intercepted water storage) and does not adequately represent the summertime use of deeper RZSM storage for T. In this way, the land surface errors illustrated in Fig. 2b ensure that, even accurately represented, JJA deficits in P will lead to an exaggerated reduction in JJA CMIP6 ET estimates. Therefore, among all three hypotheses considered here, only the land-dominant hypothesis (i.e., that land physics errors in CMIP6 lead to underestimated T/ET) is consistent with the observed positive JJA P-ET bias (Fig. 2).

Finally, CMIP6 T/ET bias can also be illustrated using RS-retrieved SM–ET coupling strength (SECS, i.e., the debiased Spearman correlation between weekly ET and surface SM RS retrievals, see “Methods”). As mentioned above, soil evaporation is controlled mainly by near-surface SM levels, while T is determined by slower-varying RZSM. Therefore, if total ET is dominated by soil evaporation (i.e., a low T/ET ratio), ET temporal dynamics will be highly consistent with that of surface SM—resulting in large (positive) SECS values^32^. Indeed, Fig. 1f shows that CMIP6 ESMs exhibit a clear positive SECS bias in the CUS that is spatially co-located with a negative bias in T/ET (Fig. 1e). This, again, supports our hypothesis that CMIP6 projections rely excessively on shallow surface SM to support CUS JJA ET and, as a result, underestimate JJA T/ET and ET while overestimating SECS.

### Inter-model relationship between T/ET and Tmax warm biases

**Fig. 3:**
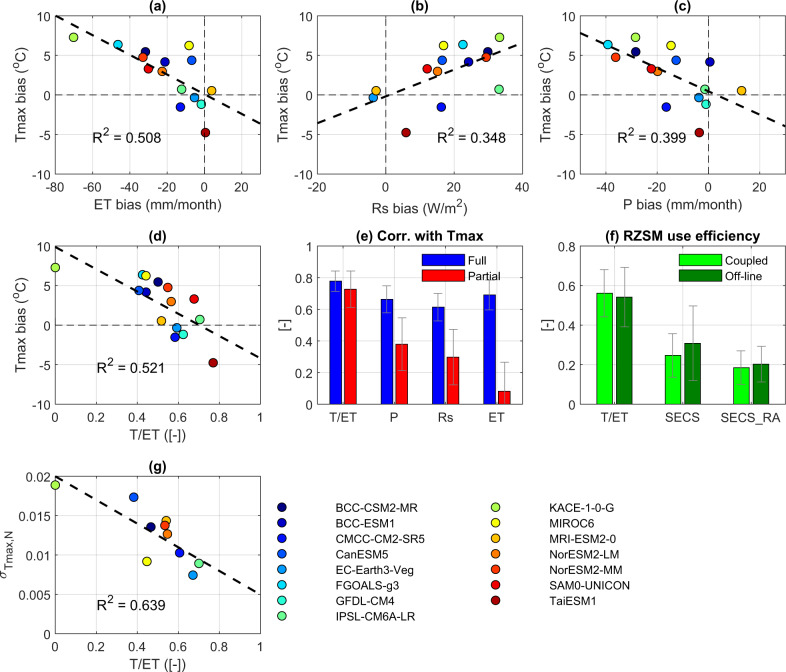
Analysis of inter-model CMIP6 variability. Inter-model variability of spatially averaged 2000–2014 June-July-August (JJA) 2-m summertime daily maximum temperature (Tmax) biases as a function of: evapotranspiration (ET, **a**), net shortwave radiation (Rs, **b**), precipitation (P, **c**) biases and modeled transpiration to ET ratio, (T/ET, **d**) within the CUS (central contiguous United States, see Fig. 1 for geolocations). **e** The full-rank correlation and partial-rank correlation of Tmax bias with T/ET, P, Rs and ET biases, where error bars represent the standard deviation of a 1500-member bootstrapping analysis. **f** The spatial-temporal mean of CUS T/ET and SECS (soil moisture—ET coupling strength) values from three CMIP6 Earth System Models (EC-Earth3-Veg, IPSL_CM6A-LR and MIROC6) and four reanalysis systems based on coupled and off-line modeling. Error bars in (f) capture the inter-model range of variables for each model group. **g** Normalized high-emission scenario (SSP585) projected 2020–2050 detrended JJA monthly Tmax standard deviations (see Methods) as a function of JJA T/ET.

The above analysis confirms that CMIP6 T/ET estimates are biased low in the CUS region—suggesting that seasonal JJA P deficits will excessively reduce ET (see also Fig. 2) and positively bias Tmax in the CMIP6 ESMs. This hypothesis is supported by Fig. 3, which demonstrates that the magnitude of the JJA Tmax bias in individual CMIP6 ESMs is strongly anti-correlated with mean JJA T/ET values (Fig. 3d). Regression results in Fig. 3d suggest that a T/ET value of 0.70 is required to achieve unbiased Tmax estimates. In contrast, 11 out of 15 CMIP6 ESMs estimate CUS summertime T/ET to be less than 0.60 (Fig. 3d).

Inter-model partial correlations (see “Methods”) between Tmax and P and Rs are both lower than 0.40 and significantly less than their full-rank equivalents (Fig. 3e). This implies that the direct contribution of P and Rs bias to Tmax bias is relatively small. Likewise, the inter-model partial correlation of Tmax and ET biases is approximately zero—meaning that its contribution to Tmax variability is mainly from compounding land/atmospheric factors. In strong contrast, the very small difference between the sampled full (0.78 ± 0.11) and partial correlation (0.71 ± 0.21) between T/ET and Tmax biases implies that CMIP6 Tmax bias is due predominately to the land surface’s direct regulation of T/ET partitioning.

These statistical findings are consistent with coupled/off-line model comparisons (Fig. 3f) illustrating that T/ET and SECS biases in off-line simulations (with relatively unbiased meteorological forcing data) are larger than corresponding biases in coupled ESMs. Once again, this implies that CMIP6 T/ET and SECS biases originate from errors in land physics and do not simply reflect the passive propagation of atmospheric biases through the land component of an ESM.

## Discussion

A notable CUS summertime (JJA) warm-and-dry bias persists in the latest generation of CMIP6 ESMs (Fig. 1). Due to strong land-atmosphere coupling in the CUS region^49^, differentiating the relative importance of atmospheric- and land-modeling errors for this bias is challenging. As a result, the dominant factors controlling the CUS JJA warm bias in CMIP6 ESMs remain unclear. Here, we provide multiple independent lines of evidence that highlight the dominant role of land model errors—particularly biases related to T/ET partitioning. Note that the contribution of T/ET bias to local land-atmosphere coupling has not been explicitly identified, or compensated for, in recent ESM versions. This may explain why CUS CMIP Tmax biases have persisted across generations of model development.

Negative T/ET biases in CMIP6 ESMs have direct implications for projected water cycle and climate change under elevated CO_2_ conditions. First, both the magnitude and sign of projected runoff changes are determined by ESM T/ET levels^50^. In addition, underestimated T/ET ensures that CMIP6 projections rely excessively on canopy-interception and surface SM storage states to supply JJA ET flux and, therefore, overestimate JJA air temperature rise due to their relative neglect of T^51^. Likewise, the inability to fully leverage RZSM storage (to supply JJA ET) suggests that CMIP6-projected Tmax is overly sensitive to normal seasonal and inter-annual variations in both P and Rs^36,37^. This hypothesis is supported by high-emission-scenario (SSP585) CMIP6 projections, which show that (ESM-specific) relative 2020–2050 JJA Tmax monthly variability with respective to P and Rs is highly sensitive to mean T/ET levels (Fig. 3g).

Based on these findings, addressing land model errors regulating T/ET partitioning should be a key priority for reducing CMIP6 regional uncertainties and biases. The simplistic representation of agricultural trends in historical ESM simulations may lead to the underestimation of ecosystem productivity/biomass and, therefore, T/ET in the CUS region. However, spatially consistent negative biases in CMIP6-estimated leaf area index (LAI) are not identified in the CUS region (Supplementary Fig. 8). In addition, increasing LAI has only a limited impact on removing T/ET bias in the CUS^32^. Therefore, underlying ESM T/ET error is most likely attributable to shortcomings in ESM representation of land surface water and energy balance processes. These shortcomings potentially include: inadequate rootzone soil water storage capacity in agricultural areas^52^, neglected lateral water flux between ESM grid cells^33,34^, neglected irrigation processes^53^, the incorrect representation of soil evaporation^32^ and structural uncertainties in stomatal conductance modeling^54^. Resolution of any of these issues could, in principle, increase the physical realism of soil water extraction by vegetation roots for JJA T. The exact land source of T/ET bias is likely to be model-specific. However, recent work illustrates that LSM T/ET bias sources can be distinguished, and effectively addressed, via the joint consideration of RS retrievals and off-line land modeling^32^. Such land-based approaches are key for reducing uncertainties in ESM projections of climate and water cycle change.

## Methods

### CMIP6 model data

Monthly mean screen-level (2 m) daily maximum air temperature (Tmax), net shortwave radiation (Rs), precipitation (P), rootzone SM (i.e., soil water content (m^3^/m^3^) of the top 50 cm soil layer, vertically integrated from CMIP6 SM profile estimates; RZSM) and evapotranspiration (ET) time series are taken from 15 historical CMIP6 ESM simulations (see Table S1 for model details) between 1 January 2000 to 31 December 2014 and resampled onto a 1-degree spatial grid via bilinear interpolation. Likewise, daily surface SM (i.e., soil water depth for the top 10 cm of the soil column) and ET from the same period and set of ESMs, are averaged into weekly mean values (to be consistent with the temporal resolution of SECS inputs, see below) and resampled onto a 1-degree spatial grid to provide model-specific estimates of surface SM versus ET coupling strength (SECS, see below for details). Resulting model-specific SECS maps are then averaged across all 15 ESMs to represent CMIP6-mean SECS. Finally, CMIP6 projections during 2020–2050 based on high-emission scenario (SSP585) results are collected from ten available ESMs (see Table S1) and used to examine inter-annual variability in future climate projections. Key CMIP6 ESM results based on multi-model means versus medians are mutually consistent (Supplementary Figs. 9 and 10).

Off-line LSM simulations (denoted as CMIP6_OFL) are collected from the CMIP6 “Land-hist” dataset^55^. CMIP6_OFL simulations use the same underlying land physics as coupled historical CMIP6 simulations. However, their meteorological and radiative forcing data are instead derived from observational datasets compiled by the third Global Soil Wetness Project (GSWP3^56,57^). GSWP3 land forcing data are generated by the dynamic downscaling of the 20th Century Reanalysis product^58^ and bias corrected using ground observations^55^. To date, only the EC-Earth-Veg3, IPSL-CM6A-LR, and MIROC6 ESMs have contributed CMIP6 Land-hist simulations containing the entire suite of data products required for our analysis. Tmax, Rs, P, SM, and ET estimates acquired from Land-hist versions of these three ESMs are processed using the resampling procedure described above.

### RS-based SECS map

Soil moisture-evapotranspiration coupling strength (i.e., SECS) is estimated based on the approach outlined in Lei et al.^59^. Specifically, we quantify SECS as the squared-rank correlation between warm-season (July to August for areas north of 35.5°N and May to October otherwise) RS-based surface SM and ET time series. Note that all RS retrievals contain random errors that tend to negatively bias RS-based SECS values. To correct for this spurious bias, triple collocation is used to estimate the error variance of each SM and ET product using three independent SM (or ET) datasets. These estimated SM (or ET) error variances are subsequently used to correct the original RS-based SECS estimates and generate unbiased global SECS maps. Key input datasets for the SECS map include the: passive European Space Agency Climate Change Initiative SM product;^60^ Advanced Scatterometer SM product^61^; Atmosphere Land Exchange Inverse ET product based on both thermal infrared imagery^41^ and Ka‐band brightness temperature^62^ and SM and ET products acquired from four different off-line Global Land Data Assimilation System LSMs^63^. According to the temporal resolution of the input RS products, the SECS map is estimated based on weekly ET and SM averages.

These RS-based, SECS estimates are then compared to comparable diagnostic statistics sampled directly from the CMIP6 and CMIP6_OFL weekly surface SM and ET products to estimate ESM SECS bias. To maximize the generality of our coupled and off-line modeling comparisons, SM–ET correlations based on reanalysis systems (denoted as “SECS_RA”) from the National Centers for Environmental Prediction (NCEP), Global Modeling and Assimilation Office (GMAO), National Center for Atmospheric Research (NCAR) and European Centre for Medium-Range Weather Forecasting (ECMWF) centers are also collected from a previous analysis^38^.

Given historical variations in the availability and quality of RS products, the SECS benchmark is based on SM and ET retrievals collected between 2007 and 2014—instead of the 2000 and 2014 historical period used for CMIP6 results. However, Supplementary Fig. 11 demonstrates that CMIP6-estimated SECS values sampled from the two periods are highly consistent.

### Reference datasets

Gauge-based 0.25-degree, daily, screen-level Tmax and P analyses are collected from the Climate Prediction Center (CPC) from 2000 to 2014. Given the high spatial density of rain gauges within CONUS, CPC observations are ideal for independently quantifying CMIP6 Tmax and P biases.

The ERA5 shortwave solar radiation product is known to be superior to other existing estimates^64^. Therefore, the ERA5 0.25-degree, hourly Rs reanalysis product between 2000 and 2014 is used as a reference dataset for all CMIP6 Rs simulations.

Flux-tower observations have limited spatial coverage within the CUS region (Supplementary Fig. 1). However, compared with flux-tower observations, ERA5 and FluxCOM ET estimates generally have a lower bias than other existing ET reference products (Supplementary Fig. 1). Therefore, the daily, 0.5-degree FluxCOM ET (RS + METEO) product^41^, available from 2001 to 2010, is used as a reference for CMIP6 ET evaluations. In FluxCOM, machine learning techniques are trained to capture ET time series with multi-source RS data at 224 global flux-tower sites. Note that FluxCOM ET estimates are not based on water balance considerations and are therefore assumed to be relatively independent of corresponding CMIP6 ET estimates. However, the FluxCOM ET reference data is only available from 2001 to 2010, which does not perfectly match our CMIP6 historical period (2000 to 2014). To examine the sensitivity of CMIP6 ET bias results to ET product selection, and exact historical sampling period, key results are duplicated using 2000 to 2014 ERA5 ET results as an alternative ET reference (see Supplementary Fig. 4). Although ER5 is a model-based product, its SM estimates are adjusted to match screen-level air temperature and humidity observations via data assimilation. Hence, ERA5-based ET estimates do not suffer from the same CUS summertime ET biases afflicting ESMs^52^—see Supplementary Fig. 1.

The 0.25-degree Global Land Evaporation Amsterdam Model (GLEAM) product has been frequently applied in diagnostic T/ET studies^47,65^. Therefore, this study uses T/ET estimates based on the 2000–2014 monthly GLEAM product as a reference benchmark. Naturally, GLEAM-based T/ET estimates are not free from error. Therefore, the potential impact of GLEAM reference bias on our findings is discussed in the “Results” section above.

In-situ profile observations from the United States Department of Agriculture Agricultural Research Service (USDA ARS) Little Washita watershed network are collected and vertically averaged into bulk (surface to 50 cm) RZSM values. Watershed-scale RZSM values are based on spatial averaging of point-scale RZSM estimates acquired from ~20 sensors scattered across the Little Washita watershed to minimize SM spatial representativeness errors^66^. The USDA ARS Little Washita network is located in an area of the CUS region where CMIP6 ESMs demonstrate significant JJA Tmax and ET biases (see Fig. 1a, b). In addition, point-scale sparse SM profile observations across the CUS region are also collected from the International Soil Moisture Network (ISMN)^67^ and used to further evaluate CMIP5 ESM RZSM estimates. Following manual quality control conducted for a previous study^40^, 27 such sites with consistent SM profile observations are available across the CUS region with coverage between 2000 and 2014. Evaluation results based on these sparse sites are shown in Supplementary Fig. 3.

### Partial correlation analysis

Partial correlation analysis is applied to disentangle the inter-model contributions of land and atmospheric errors to Tmax bias. The partial correlation of Tmax and T/ET (denoted as P_T/ET_), for example, treats P, Rs, and ET as holding variables to statistically isolate their impact. As such, P_T/ET_ reflects only land physics impacts on Tmax via T/ET. Therefore, the difference between P_T/ET_ and the full-rank T/ET versus Tmax correlation captures the impact of atmospheric errors, i.e., if Tmax is entirely controlled by P and Rs, P_T/ET_ will be zero. In contrast, if T/ET is dominated by land errors, P_T/ET_ will equal the full-rank correlation of T/ET and Tmax. Analogous partial correlation analysis is also performed to derive the partial correlation of Tmax with ET, P and Rs. For simplicity, we focus only on absolute correlation coefficients.

### Normalized air-temperature variability

Land impacts on projected JJA daily Tmax variability (captured by monthly JJA standard deviations during the 2020–2050 period, denoted as ${\sigma }_{{{{{\rm{T}}}}}{\max }}$) are examined. To isolate the impact of seasonal and long-term trends, detrended anomalies are used. Additionally, ${\sigma }_{{{{{\rm{T}}}}}{\max }}$ is normalized by the variability of detrended P and Rs variability (i.e., normalized Tmax variability, ${\sigma }_{{{{{\rm{T}}}}}{\max },N}=\frac{{\sigma }_{{{{{\rm{T}}}}}{\max }}}{{\sigma }_{{{{{\rm{P}}}}}}{\sigma }_{{{{{{\rm{Rs}}}}}}}}$) to reflect the sensitivity of Tmax variability to P and Rs variability.

## Supplementary information

Supplementary Information

## Data Availability

The CMIP6 data are available at: https://www.wcrp-climate.org/wgcm-cmip/wgcm-cmip6. ISMN soil moisture data are available at: https://ismn.geo.tuwien.ac.at/en/. FluxCOM and GLEAM ET data can be found at: http://www.fluxcom.org/CF-Download/ and https://www.gleam.eu/, respectively. CPC data are available at https://psl.noaa.gov/data/gridded/data.unified.daily.conus.html. ERA5 data can be found at: https://www.ecmwf.int/en/forecasts/datasets/reanalysis-datasets/era5. The processed data for CMIP6 evaluation are available at: https://zenodo.org/record/5745862.YafAD7pOk2w.

## Code availability

Codes for data processing, evaluation and visualization are available at: https://zenodo.org/record/5745862.YafAD7pOk2w.
