# Supplementary material for: Land transpiration-evaporation partitioning errors responsible for modeled summertime warm bias in the central United States
Source: Nat Commun. 2022 Jan 17;13:336. doi: 10.1038/s41467-021-27938-6 (PMC8764074; doi:10.1038/s41467-021-27938-6)
Supplement: Supplementary file 1 — Supplementary Information [file 41467_2021_27938_MOESM1_ESM.pdf]

Land Transpiration-Evaporation Partitioning Errors Responsible for Modeled Summertime Warm Bias in the Central United States

Jianzhi Dong[1, 2], Fangni Lei[3] and Wade Crow[2]

1. Department of Civil and Environmental Engineering, Massachusetts Institute of Technology, Cambridge, Massachusetts, USA
2. USDA Hydrology and Remote Sensing Laboratory, Beltsville, Maryland, USA
3. Geosystems Research Institute, Mississippi State University, Starkville, Mississippi, USA

1. CMIP6 Earth System Models

Table S1: The native spatial resolution of Earth System Models (ESMs) utilized in the analysis and the availability of their CMIP6 "Historical", "Land-hist" and "SSP585" experiments (as of summer 2021).

Model	Resolution (degrees)	Historical	Land_hist	SSP585
BCC-CSM2-MR	1.25 × 1.25	√		√
BCC-ESM1	2.81 × 2.81	√		
CMCC-CM2-SR5	1.25 × 0.94	√		√
CanESM5	2.81 × 2.81	√		√
EC-Earth3-Veg	0.70 × 0.70	√	√	√
FGOALS-g3	2.00 × 2.00	√		
GFDL-CM4	1.25 × 1.00	√		
IPSL-CM6A-LR	2.50 × 1.26	√	√	√
KACE-1-0-G	1.87 × 1.25	√		√
MIROC6	1.40 × 1.40	√	√	√
MRI-ESM2-0	1.12 × 1.12	√		√
NorESM2-LM	2.50 × 1.89	√		√
NorESM2-MM	1.25 × 0.94	√		√
SAM0-UNICOM	1.25 × 0.94	√		
TaiESM1	1.25 × 0.94	√		

2. Ground-based evaluation of ET products

This section evaluates five well-known ET reference products and determines the least-unbiased product(s) for CMIP6 evaluation. Ground-based Fluxnet2015 ET observations that have been quality controlled by a previous ET analysis[63] are used. Additionally, only flux towers within the middle of North America, where CMIP6 estimates have strong warm biases, are considered (Figure S1a). Observations from the resulting 14 flux towers are linearly averaged to create a 1-degree spatial resolution ET product applied to evaluate bias in each reference ET product. On average, FluxCOM is the least-biased reference ET product with a mean positive bias of 11.5 mm/month, followed by ERA5 (16.9 mm/month). Note that the site-to-site differences of flux tower-based ET estimates within a 1-degree grid-cell range from 6.3 to 29.1 mm/month – suggesting that FluxCOM and ERA5 biases are generally within the range of observational uncertainties.

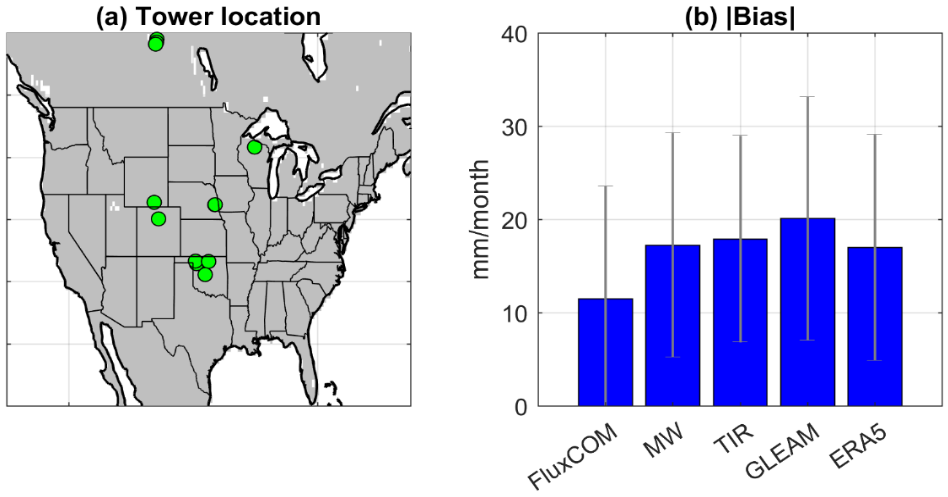

Figure S1: The spatial distribution of the 14 flux tower sites considered (a) and the absolute biases of five RS-based and reanalyzed ET products relative to flux tower observations across these sites (b). Error bars in part (b) capture the standard deviation of sampled biases across all grid cells containing flux towers.

3. Horizontal temperature advection

Temperature advection impacts are investigated using hourly Modern-Era Retrospective analysis for Research and Applications, Version 2 (MERRA-2) JJA 2012 results collected from GES DISC (https://daac.gsfc.nasa.gov/). Figure S2a demonstrates that hourly temperature advection (A) is, on average, one to two orders of magnitude smaller than the hourly air temperature change rate (denoted as dT/dt). This is evident for Figure S2b, which shows that hourly dT/dt is up to 5 K/h at local noon while hourly A is almost exclusively less than 0.03 K/h. Therefore, based on simple heat-balance considerations, A appears to have a limited impact on the net diurnal heating of the lower atmosphere and thus on Tmax itself.

The true impact of A in Figure S2b may be masked by diurnal solar radiation cycles and emerge only when examined at a daily time scale. To examine this possibility, we estimated the day-to-day variance of Tmax explained by A. Such explained variance is close to zero for most pixels in the CUS region – suggesting a minor role for temperature advection at the daily time scale.

It should be noted that these analyses are also consistent with land-water balance results shown earlier in Figure 1 of the main text. If A truly dominated Tmax bias, it would also enhance evaporative demand and thus yield positively biased ET and negatively biased RZSM (i.e., depleted rootzone water storage via enhanced ET). However, neither of these signals are observed in Figure 1 – which, once again, implies a relatively small role for A.

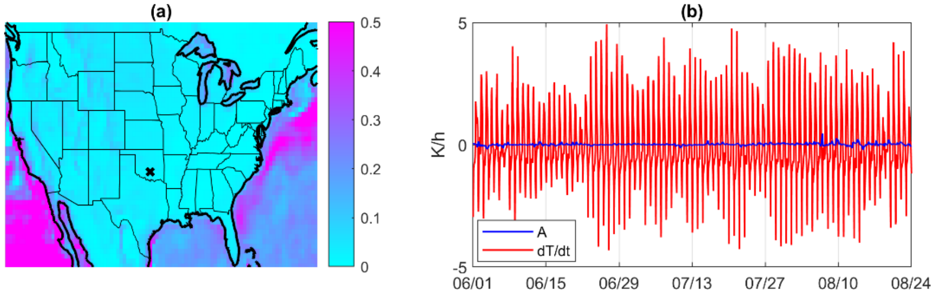

Figure S2: The ratios of daily maximum horizontal temperature advection (i.e., A) and air temperature change rate (i.e., dT/dt) averaged across 2012 JJA months (a). The time series of hourly A and dT/dt from an illustrative pixel in Oklahoma (see the black cross in part (a) for geolocation) are shown in (b).

4. Evaluation of CMIP6 RZSM drying rates using sparse SM observations

Given that CMIP6-modeled water balance variables are relatively unbiased in May (see Figure S4), we define the relative JJA RZSM depletion rate as $RZSM_{JJA}/RZSM_{May}$ (denoted as RZSMns) where subscripts indicate the temporal averaging domain applied to RZSM values. As shown in Figure S3, the sign of biases in CMIP6 JJA RZSM drying rates (i.e., CMIP6 RSZMns minus observed RZSMns values) are geographically mixed. Moreover, a paired T-test based on these 27 sites indicates that observed differences between modeled and observed RZSMns values are not significantly different.

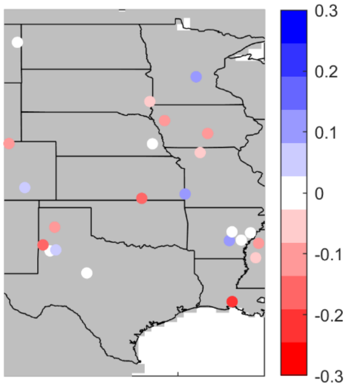

Figure S3: Biases in CMIP6 RZSM drying rates during 2000 to 2014 JJA (RZSMns) evaluated using 27 ground-based RZSM measurement sites. Blue and red shading indicate that CMIP6 RZSMns is biased lower and higher than ground observations, respectively.

5. Impact of reference ET product choice

Figure S4 shows that monthly CMIP6 ET biases evaluated using FluxCOM and ERA5 ET reference products are generally consistent. It also confirms that CMIP6 ET biases calculated using both the FluxCOM and ERA5 ET references exceed P biases and 3-monthly averaged P biases (denoted as P3) – most notably in July and August. This demonstrates that our conclusion that ET biases are larger (i.e., more highly negative) than corresponding P biases is robust across different ET reference datasets. In addition, the observed P - ETr relationship based on the use of ERA5 as the reference ET dataset (Figure S5) qualitatively matches earlier results based on the use of FluxCOM as the ET reference (Figure 2).

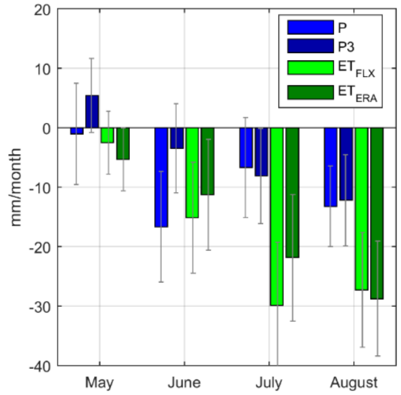

Figure S4: The monthly bias of CUS-spatially averaged P and P3 (3-monthly averaged P where June P3, e.g., is average-daily P during April-May-June). ET is based on the use of both FluxCOM (ET_{FLX}) and ERA5 (ET_{ERA}) estimates as the reference ET product.

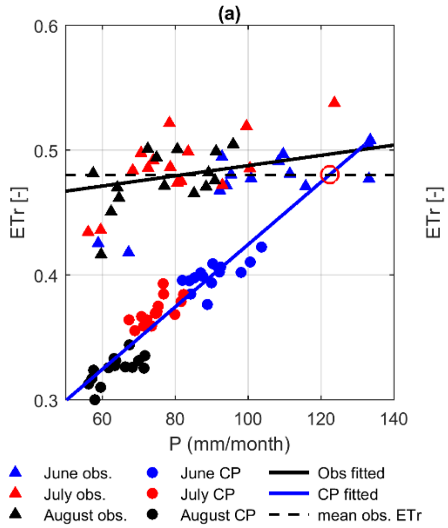

Figure S5: (a) Observed and CMIP6 modeled CUS monthly mean ETr as a function of monthly mean precipitation (P) during 2000–2014 June-July-August (JJA) periods. The large open red circle in (a) captures the intersection of the extrapolated CMIP6 regression line with the monthly-mean-observed ETr line. Part (b) is same as (a) but for 3-model-averaged (EC-Earth3-Veg, IPSL-CM6A-LR and MIROC6) CMIP6 (denoted as CP) and CMIP6_OFL (CMIP6 offline simulation, denoted as OFL) results. In contrast to Figure 2, refrence ET values are based on ERA5 estimates.

6. ETr-P relationships for individual ESMs

Figure S6a shows that 13 of the 15 individual ESMs overestimate the sensitivity of ETr to P. The MIROC-6 and MRI-ESM2-0 ESMs represent the two exceptions to this tendency. MIROC-6 model-based ETr versus P sensitivity is comparable to that of observations, but its magnitude of ETr is biased substantially low for all P ranges (green line in Figure S6a). Relative to other ESMs, the MRI-ESM2-0 ETr (red line in Figure S6a) versus P relationship is most comparable to that of observations, and, as a result, its ET and Tmax biases are also relatively unbiased (Figure 3). In addition, Figure S6b confirms our earlier finding in Figure 2 that the ETr versus P relationship is dominated by land physics, instead of atmospheric modeling errors.

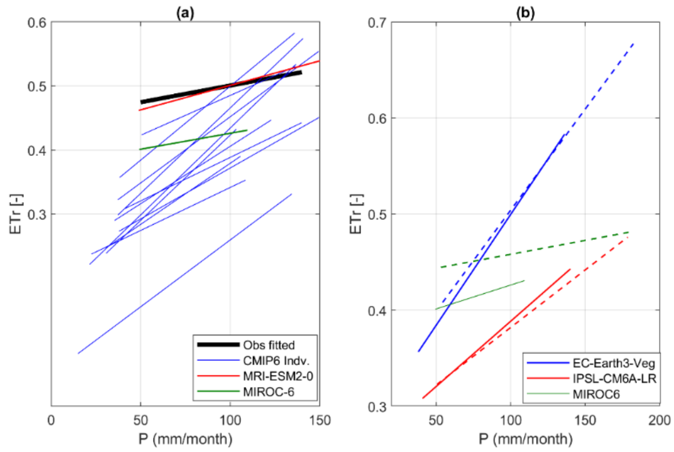

Figure S6: (a) Observed and CMIP6 modeled CUS monthly mean ETr as a function of monthly mean precipitation (P) during 2000–2014 June-July-August (JJA) periods for individual ESM models. Solid and dashed lines in part (b) are fitted lines for CMIP6 and CMIP6_OFL simulations, respectively.

7. ESM performances over dry-wet transitional climate zones

According to GLEAM estimates, both T/ET and ET/PET (potential ET) ratios increase from west to east in the CUS region, which is consistent with the known geographic distribution of vegetation density and aridity (Figure S7). By excluding energy-limited areas (defined as ET/PET > 0.95) and land grid cells with low vegetation contributions to ET (defined as T/ET < 0.7), the remaining land grid-cells are spatially consistent with areas where the CMIP6 ESMs present their largest T/ET and Tmax biases (i.e., compare Figure S7c and Figure 1e).

These results suggest the general difficulty of capturing complex vegetation-atmosphere interactions in ESMs over dry-wet transitional climate zones. In arid/barren regions (e.g., the western US), T/ET is very low. As a result, absolute negative biases in modeled T/ET are typically small. On the other hand, the eastern US is typically energy-limited – meaning that vegetation/soil water stress errors have a limited impact on T/ET partitioning. As a result, land surface modeling errors in CMIP6 ESMs (related to soil/vegetation water stress) are most notable in the CUS region since the region is characterized by a combination of frequent transitions between dry and wet JJA conditions and significant T contributions to ET.

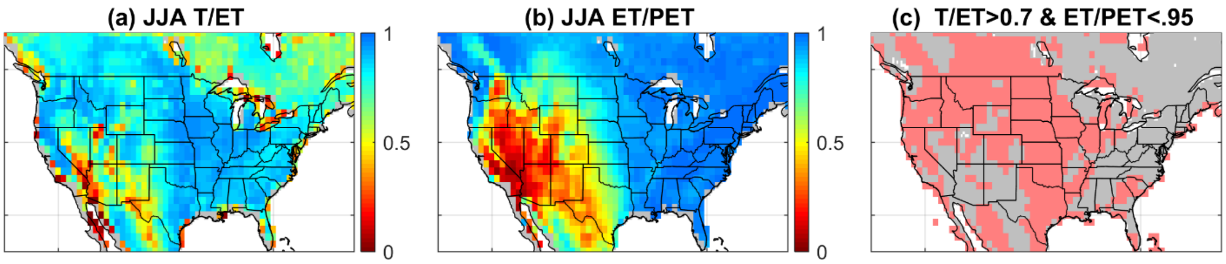

Figure S7: GLEAM-estimated T/ET (a) and ET/PET (b) during 2000–2014 summer months (JJA). Red shading in (c) denotes land grids with T/ET > 0.7 and ET/PET < 0.95.

8. Evaluation of CMIP6-modeled leaf areas index (LAI)

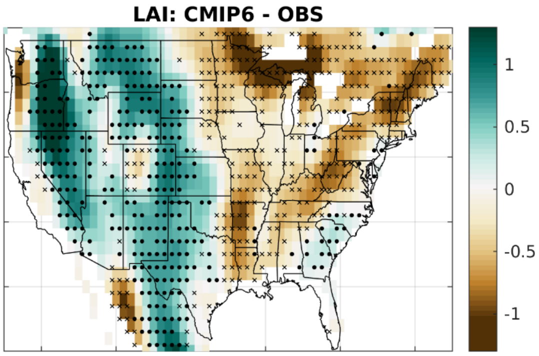

Figure S8. Comparison of CMIP6-modeled and RS-based JJA monthly mean LAI. Dotted and crossed areas are land pixels where at least two-thirds of the individual ESMs are biased high and low, respectively, compared to remotely sensed LAI.

9. Evaluation based on multi-model median values

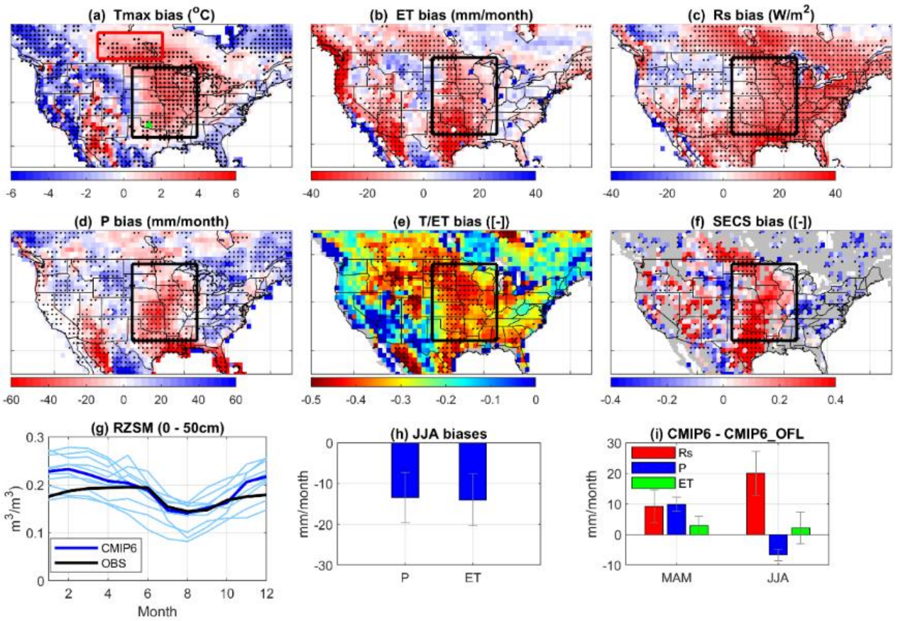

Figure S9: 2000–2014 CMIP6 biases within JJA: Tmax (a), ET (b), net shortwave radiation (Rs, c), Precipitation (P, d), Global Land Evaporation Amsterdam Model (GLEAM) evaluated transpiration (T) to ET ratio (T/ET, e) and soil moisture – ET coupling strength (SECS, f) estimates. Dotted grids indicate that two-thirds of individual Earth System Model (ESM) biases are consistent in sign (in (a)-(d) and (f)) or two-thirds of ESMs are 0.3 lower than GLEAM-based T/ET (in (e)). (g) Monthly-mean CMIP6 RZSM estimates compared to a dense soil moisture network (OBS) within the state of Oklahoma (see the green triangle in part a). The thin and thick blue lines represent individual ESMs and their multi-model means, respectively. (h) June-July-August (JJA) CMIP6 P and ET biases averaged across 15 CMIP6 ESMs. Error bars denote their inter-model standard deviations. (i) The mean difference of CMIP6 and CMIP6_OFL (CMIP6 offline simulations) based Rs, P and ET estimates for three individual ESMs during both March-April-May (MAM) and JJA. Error bars represent the range of CMIP6 and CMIP6_OFL differences. Multi-model medians (as opposed to multi-model means in Figure 1) to summarize results across CMIP6 ESMs.

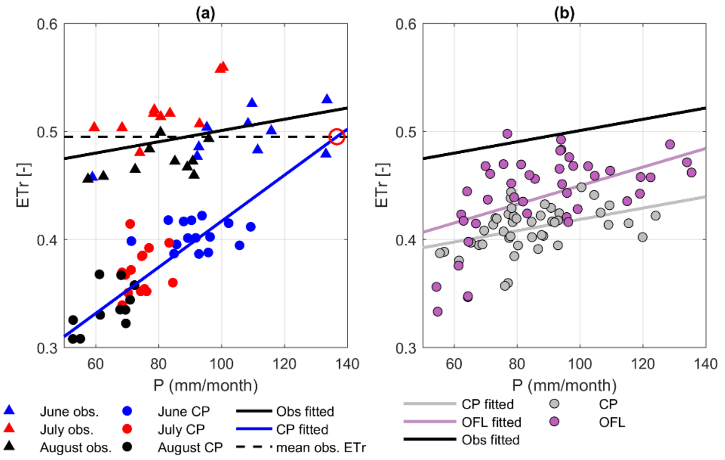

Figure S10: (a) Observed and CMIP6 modeled CUS monthly mean ETr as a function of monthly mean precipitation (P) during 2000–2014 June-July-August (JJA) periods. The large open red circle in (a) captures the intersection of the extrapolated CMIP6 regression line with the monthly-mean-observed ETr line. Part (b) is same as (a) but for 3-model-averaged (EC-Earth3-Veg, IPSL-CM6A-LR and MIROC6) CMIP6 (denoted as CP) and CMIP6_OFL (CMIP6 offline simulation, denoted as OFL) results. ET values are based on FluxCOM estimates. Multi-model medians (as opposed to multi-model means in Figure 2) to summarize results across all CMIP6 ESMs.

10. SM-ET coupling strength (SECS) sampled on different temporal periods

Due to temporal variations in remote sensing (RS) data availability, benchmark SECS values are sampled using SM and ET retrievals collected from 2007 to 2014. In contrast, ESM-based SECS values are sampled from the entire 2000 to 2014 historical period. To investigate the impact of this temporal mismatch, Figure S11 compares ESM-based SECS sampled using both 2007–2014 and 2000–2014 CMIP6 model outputs. Results demonstrate that SECS values from these two historical sampling periods are highly consistent. Therefore, benchmark (RS-based) SECS biases sampled in the (shorter) 2007 to 2014 period are assumed to be representative of bulk conditions during the (longer) 2000 to 2014 historical period.

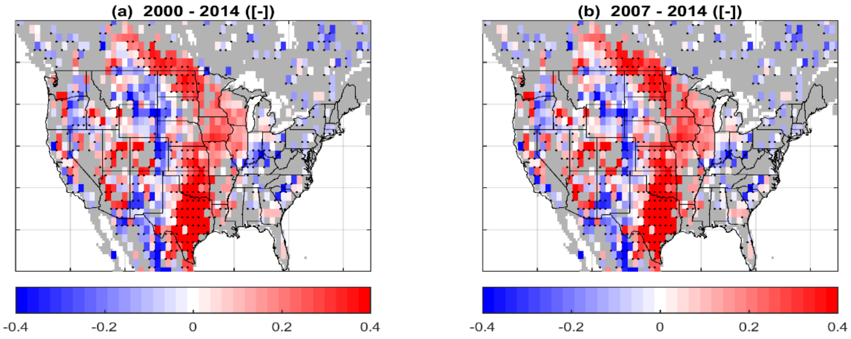

Figure S11: CMIP6 ESM mean SM-ET coupling strength (SECS) sampled during the 2000–2014 (a) and 2007–2014 (b) warm seasons.
